# Myofibroblastoma of the Breast: A Morphologic and Immunohistochemical Study of Three Cases

**DOI:** 10.30699/IJP.2021.138647.2520

**Published:** 2020-07-06

**Authors:** Hiva Saffar, Dorna Motevalli, Nasibeh Seirfar, Mahsa Ebrahimi, Perikala Vijayananda Kumar, Farid Kosari, Hedieh Moradi Tabriz, Sadaf Naderi, Golsa Shekarkhar

**Affiliations:** 1 *Department of Pathology, Shariati Hospital, Tehran University of Medical Sciences, Tehran, Iran*; 2 *Department of Pathology, Shiraz University of Medical Sciences, Shiraz, Iran *; 3 *Department of Pathology, Tehran University of Medical Sciences, Tehran, Iran*; 4 *School of Medicine (MBBS), University of Central Lancashire, Preston, United Kingdom*

**Keywords:** Immunohistochemistry, Myofibroblastoma, Variants

## Abstract

Myofibroblastoma (MFB) of the breast is an uncommon entity of benign spindle neoplasms of the breast. This tumour possesses a broad spectrum of histomorphological patterns. Distinguishing of myofibroblastoma variants from malignant mimics of this benign neoplasm is essential for pathologists to avoid further invasive surgical procedures. In this article, we report the clinical, morphological, and immunohistochemical features of three cases, including two females and one male patient with mammary myofibroblastoma with emphasis on the histomorphological findings. As there is not yet enough information about MFB, more reports of MFB are still required to more clarify the pathogenesis and potential predisposing factors of this rare type of breast tumours.

## Introduction

Mammary myofibroblastoma (MFB) is an uncommon type of benign spindle stromal tumours described to occur in the breast (1). Sixteen cases were first described by Wargotz* et al.* in 1987 (2). The first case series was reported mostly in male patients; however, subsequent case reports revealed the occurrence of the tumour in females as well as male patients (1-4). In recent decades, the incidence of MFB has been raised, which can be due to the increased use of imaging techniques for screening purposes. MFB has been suggested to arise from mammary stromal fibroblasts with positivity for CD34 and vimentin markers. On the other hand, expression of ER, PR and AR receptors in these cells guided us to a probable role of sex steroid hormones in their pathogenesis (5). Myofibroblast cells are capable of multidirectional mesenchymal differentiation, which can further differentiate into smooth muscle, cartilage, or osseous tissue (1).

Differential diagnoses of myofibroblastoma are benign conditions including reactive processes and benign neoplasms such as nodular and proliferative fasciitis, spindle cell lipoma, neurofibroma and leiomyoma. More-over, low-grade malignant sarcomas such as spindle cell liposarcoma, low grade myofibroblastic sarcoma, and metaplastic spindle cell carcinoma also encompass the differential diagnoses of MFB (6).

Various morphologic variants of myofibroblastoma may be misdiagnosed as malignant neoplasms such as stromal sarcoma, high grade undifferentiated pleomorphic sarcoma and spindle or metaplastic carcinoma; there-fore, pathologists must be able to distinguish myofibroblastoma from other malignant mimickers and avoid over-diagnosing the malignant lesions and unnecessary invasive interventions. Herein, we report 3 cases of mammary MFB in one male patient and 2 female patients from three tertiary referral centres in southern and northern Iran.

## Case Presentation

Case 1

**Fig. 1:**
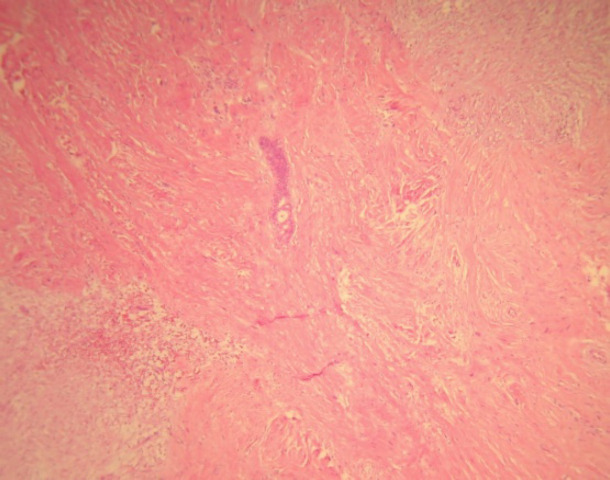
Myofibroblastoma, Collagenous/fibrous variant. Hypocellular neoplasm with a densely hyalinized stroma. Entrapped breast ductal and lobular structures are noted. Haematoxylin and Eosin (H&E) (40X magnification)

A 52-year-old woman was referred to “Shariati hospital, Tehran, Iran” due to a palpable lump in the right mammary region, she was otherwise healthy. Lumpectomy was done on one ovoid piece of well-demarcated unencapsulated soft tissue measuring 2.5x2.3x1.2 cm with a rubbery consistency. Cut sections of the specimen revealed a pinkish whorling pattern. Tumour tissue was fixed in formalin, and slides from paraffin blocks were stained with haematoxylin-eosin (H&E). The histomorphological study revealed a neoplasm with pushing border composed of ovoid to elongated cells with mild to moderate amount of eosinophilic cytoplasm, arranged in fascicles. Some multinucleated floret-like giant cells were observed. Deposition and myxoid degeneration were present in between areas of collagen. Mitotic activity was nil and entrapped breast ductal, and lobar structures were also identified (Figure 1).

**Fig. 2:**
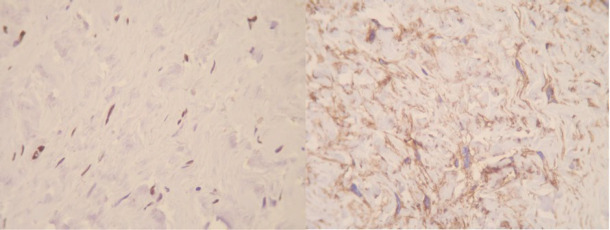
Immunohistochemical staining for progesterone receptor (PR) [left] and CD10 [right], (100X magnification)

Immunohistochemical studies revealed various tumour cells positive for progesterone receptor (PR), smooth muscle actin (SMA), CD34, and CD10. Ki-67 was positive in less than 5% of tumour cells (Figure 2).

Case 2

A 75-year-old male was referred to “Sina Hospital, Tehran, Iran”, presenting with a long-lasting history of unilateral left-sided gynecomastia without nipple discharges or retractions and had no family history of cancer. Examining the patient, a round palpable mass was detected in his left breast region. Surgical excision was performed with 1 cm free surgical margins. On gross examination, a round greyish well-circumscribed mass with a firm consistency and homogenous cut surfaces was detected. Histological sections showed a hypercellular spindle neoplasm composed of haphazardly arranged and occasionally intersecting fascicles of bland-looking spindle shape tumour cells having hyperchromatic nuclei, inconspicuous nucleoli, and eosinophilic cytoplasm. Occasional palisading and ropey-like collagen deposition were identified. No necrosis, atypia or mitosis were confirmed (Figures 4 and 5).

Immunohistochemical study showed positivity of tumoral cells for desmin, CD34, and progesterone receptor (PR) (Figure 5). 

**Fig. 3:**
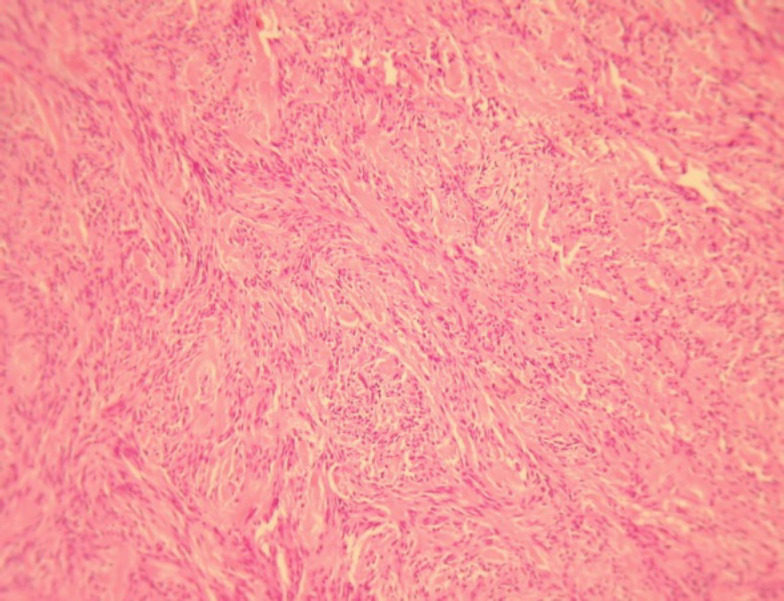
Myofibroblastoma, Classic variant, Histologic sections demonstrate haphazardly arranged rather bland-looking spindle shape tumoral cells. Haematoxylin and Eosin (H&E), (40X magnification)

**Fig. 4:**
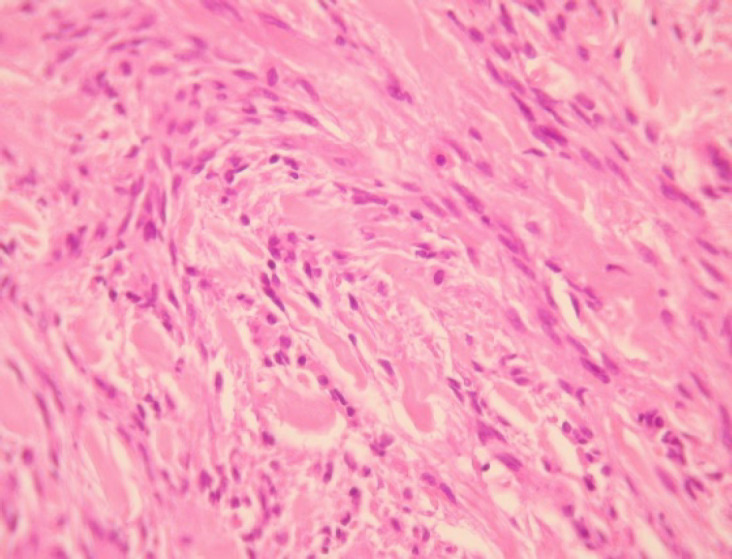
A dense neoplastic area composed mainly of neoplastic cells with fibroblast-like appearance that intermingled with collagen bundles. Haematoxylin and Eosin (H&E), (400X magnification)

**Fig 5:**
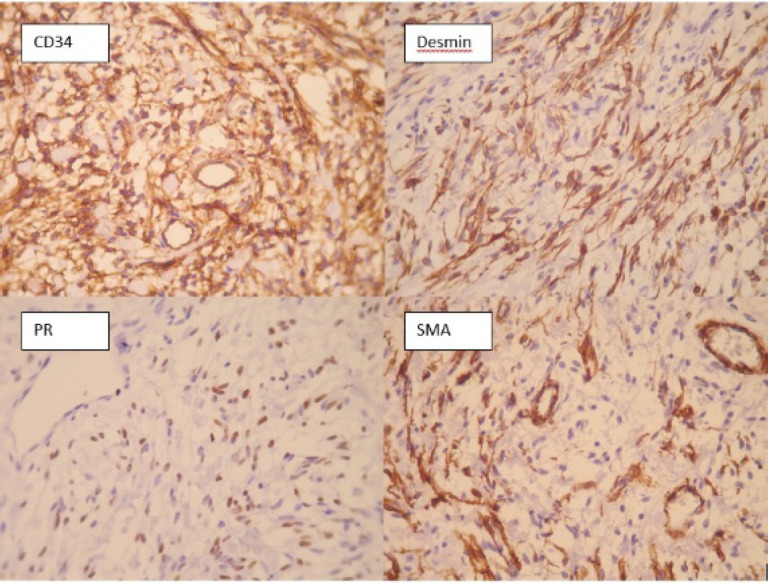
IHC staining for CD34, desmin, Progesterone receptor (PR), and α-Smooth muscle actin (SMA), (400X magnification)

Case 3

**Fig 6:**
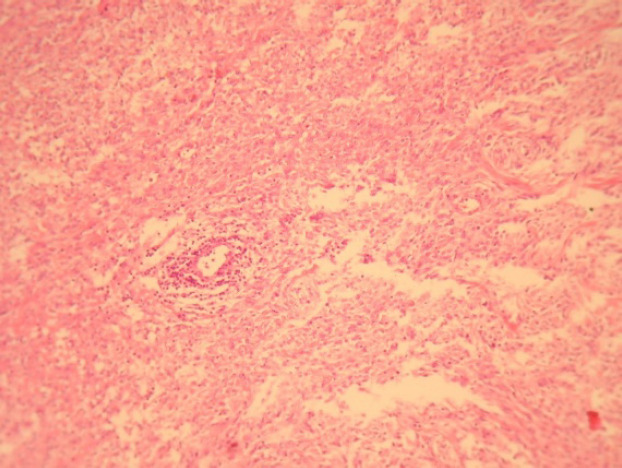
Fascicles of spindled cells with stromal hyaline depositions. Haematoxylin and Eosin (H&E), (40X magnification)

**Fig 7:**
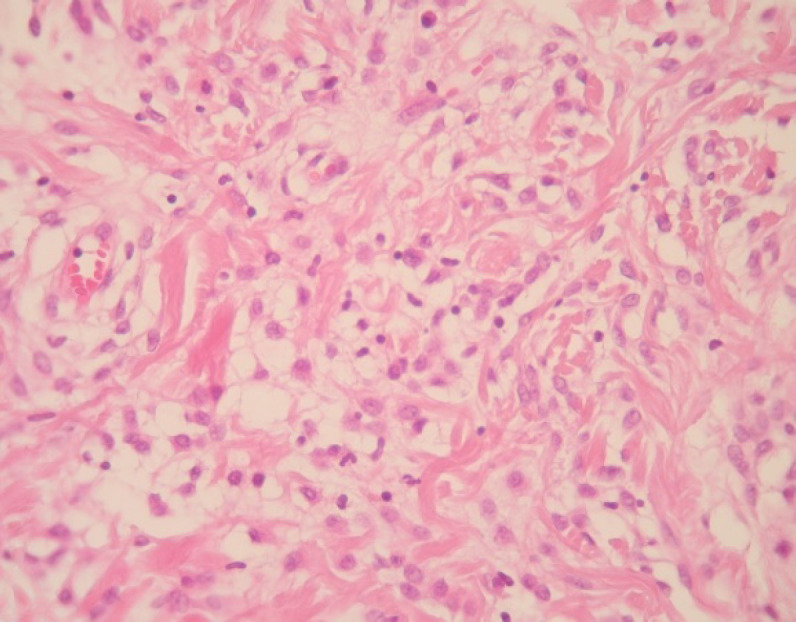
Myofibroblastoma with myxoid change plump-spindle shaped tumoral cells intermingled with collagen bundles. Haematoxylin and Eosin (H&E), (400X magnification)

The patient was a 55-year-old female who referred to “Nemazee hospital, Shiraz, Iran” with a chief complaint of left breast mass, which gradually enlarged. The intervention was done to surgically remove the mentioned mass. On gross examination, the tumour was a grey-white rubbery well-circumscribed tissue measuring 3 x 2.5 x 2 cm. Histopathology sections demonstrated a fascicular arrangement of tumoral cells with the bland-looking spindle-shaped appearance admixed with hyalinized collagen bundles with areas of myxoid changes (Figures 6 and 7). 

On immunohistochemical staining, tumoral cells were positive for CD34, and focally positive for oestrogen receptor (ER). 

## Discussion

The entity of MFB was first introduced by Toker* et al.* in 1981 (7), years later in 1987, the term “myofibroblastoma” was reported by Wargotz* et al.* (2). The first reports showed a male predilection, however, subsequent reports showed occurrence of MFB in both genders. Most cases are diagnosed in men and postmenopausal women. MFB has been known to be sporadic, but in some patients, it has been diagnosed in the setting of gynecomastia. In our study, the second case presentation, the tumour was found in a patient with gynecomastia (8, 9). MFB is currently ranked in the category of benign mesenchymal tumours with deletion of 13q14 region, which has been seen either in spindle cell lipoma or cellular angiofibroma (10). 

Grossly, MFB is reported as an unencapsulated round, oval, or lobulated mass with firm and rubbery consistency. Cut surfaces are white to greyish with whirling homogeneous appearance (1). All our cases were oval masses with rubbery consistency.

Histomorphologically, MFB is a purely mesen-chymal tumour composed of bland-looking spindle cells, which is interspersed with hyalinized collagen fibers, and sometimes foci of infiltration at the periphery which may entrap benign breast glands and adipocyte tissue (11). The tumour also shows low proliferative activity, without atypia and necrosis. Several histologic variants are mainly due to the capacity of myofibroblasts to multidirectional mesen-chymal differentiation. At least eight histologic var-iants of MFB have been discussed in the literature, including classic-type, cellular, infiltrative, epithelioid, desidualoid-like, collagenised/fibrous, lipomatous, and myxoid variants (1, 11). In our study, the first case was categorised in collagenized/fibrous variant, in which spindle cells are seen in a dense collagenized stroma. Collagen fibers formed slit-like spaces (Figure 1). The second case was a typical classic-type of myofibro-blastoma composed of bland-looking haphazardly intersecting spindle cells admixed with hyalinized collagen bundles (Figure 3, 4). The third case showed myxoid changes. (Figure 6, 7)

MFB may be misdiagnosed as malignancy depending on the clinical setting and morphological features alone. Therefore, Immunohistochemistry plays an essential role in the establishment of final diagnosis, especially regarding unusual variants. According to literature, most of the cases are positive for mesenchymal markers such as vimentin, CD34, and desmin (1). Variable expressions of smooth muscle actin, BCL2 and CD99 have been reported, and most of the reported tumours were positive for estrogen, progesterone receptor, and androgen receptor. 

In our first case presentation, IHC staining for CD34, SMA, BCL2 and Progesterone receptor were positive. (Figure 2) CD10 was also positive in some tumoral cells, which is consistent with the previous studies and has been regarded as an evidence of linking between mammary myofibroblastoma and spindle cell lipoma of soft tissue (12). S100, and cytokeratin did not stain tumoral cells, which is consistent with the previous reports (1). Other markers which were negative included desmin, beta-catenin, CD68, estrogen receptor, and CD99. Ki67 stained about 1 percent of tumoral cells. Second case was positive for desmin, CD34, and progesterone receptor. Tumoral cells also showed weakly positivity for estrogen receptor. (Figure 5) S100, SMA, BCL2, Caldesmon were negative in spindle tumoral cells and Ki 67 showed 1% proliferative index. The third case showed positivity for CD34 and focal positivity of estrogen receptor. Markers of CD68, HMB45, vimentin, Cytokeratin, and CD31 were negative. Ki 67 was positive in less than 1% of tumour cells.

## Conclusion

In conclusion, more reports of MFB are still required to more clarify the pathogenesis and potential predisposing factors of this rare type of breast tumours.
